# Patterns of Genetic and Clonal Diversity in *Myriophyllum spicatum* in Streams and Reservoirs of Republic of Korea

**DOI:** 10.3390/plants14172648

**Published:** 2025-08-26

**Authors:** Eun-Hye Kim, Kang-Rae Kim, Mi-Hwa Lee, Jaeduk Goh, Jeong-Nam Yu

**Affiliations:** 1 Nakdonggang National Institute of Biological Resources, Sangju 37242, Republic of Korea; karpo83@nnibr.re.kr (E.-H.K.); blume96@nnibr.re.kr (M.-H.L.); jdgoh@nnibr.re.kr (J.G.); 2 Southeast Sea Fisheries Research Institute, National Institute of Fisheries Science, Namhae 53085, Republic of Korea; kimkangrae9586@gmail.com

**Keywords:** aquatic macrophytes, clonal, genetic diversity, habitat type, microsatellite, *Myriophyllum spicatum*, spatial genetic structure

## Abstract

*Myriophyllum spicatum* is a globally distributed aquatic plant capable of sexual and clonal reproduction. Despite its ecological importance and biochemical potential, studies on its genetic and clonal structure in freshwater systems throughout South Korea remain limited. We investigated the genetic and clonal diversity of *M. spicatum* using 30 newly developed microsatellite markers across 120 individuals from six freshwater systems in South Korea. Overall, 148 alleles were identified, with an average polymorphism information content value of 0.530. Clonal diversity differed among populations, with the genotypes to individuals (G/N) ratio ranging from 0.200 to 1.000. Bottlenecks and clonal dominance were observed in riverine populations. High genetic differentiation (mean F_ST_ = 0.556) indicated limited gene flow, and STRUCTURE analysis revealed six distinct genetic clusters. No significant correlation was found between genetic and geographic distance, suggesting possible seed dispersal by waterfowl, particularly between adjacent populations. Genetic structure was shaped by habitat type, disturbance intensity, and reproductive strategy. Stable reservoir habitats favored sexual reproduction and higher genetic diversity, whereas disturbed river systems showed clonal dominance and reduced variation. These findings provide essential genetic insights for conservation planning and sustainable management of aquatic plant resources.

## 1. Introduction

Organisms naturally inhabit fragmented environments and adapt to the associated environmental conditions. Depending on the species, habitat characteristics influence various reproductive strategies [1,2,3,4,5]. The mode of reproduction largely determines gene transmission across time and space, making it a key factor in shaping population genetic structure [6,7,8,9].

Plants employ diverse reproductive strategies, including sexual and clonal reproduction, which significantly impact genetic diversity within populations. The balance between these two modes can vary widely depending on habitat conditions and genetic factors [10]. This balance, in turn, directly influences the genetic structure of natural populations. Clonal reproduction is expected to result in lower genetic diversity because of reduced gene flow, as it produces genetically identical offspring [11,12,13]. However, it can enhance survival by facilitating the rapid expansion of individuals and increasing reproductive success under certain conditions [14,15,16].

In freshwater ecosystems, submerged plants are crucial as primary producers and contribute to environmental stability [17,18,19]. Aquatic environments experience frequent disturbances and rapid changes, placing selective pressure on plants to adapt their growth and reproductive strategies [20,21,22]. Many aquatic plants have evolved the ability to reproduce both sexually and clonally to cope with these environmental challenges [23]. When sexual reproduction is limited because of ecological constraints, clonal reproduction becomes the dominant strategy, often favoring traits that enhance survival and environmental adaptability [24,25,26,27,28]. Clonal reproduction is particularly prevalent in wet, nutrient-poor, cold, or shaded environments [25]. Given these conditions, it is a common reproductive strategy among aquatic plants [29,30,31,32].

*Myriophyllum spicatum*, a native species in South Korea [33], is a perennial aquatic plant widely distributed across Europe and Asia, capable of thriving in diverse environmental conditions. This species prefers alkaline waters and is often found in disturbed areas affected by eutrophication [34,35,36,37]. In Europe, the United States, and South America, *M. spicatum* has been designated as an invasive species because of its aggressive reproduction and rapid spread [38,39,40,41]. It has also been identified as a dominant species in certain lakes [42,43]. The reproductive biology of *M. spicatum* contributes to its dominance. The flower stems emerge above the water surface, displaying seasonal dominance in distribution patterns. Male flowers are positioned at the top of the stem, whereas female flowers grow below during the early summer breeding season [44]. Simultaneously, auto-fragmentation occurs as flowers bloom, further enhancing its ability to spread. Seed germination occurs at water temperatures above 10 °C, providing a competitive advantage over other submerged species [45]. Although *M. spicatum* serves as a habitat for juvenile aquatic organisms, its excessive growth can lead to negative ecological impacts, such as the formation of dense canopies that limit light penetration and nutrient availability [46,47].

*Myriophyllum spicatum* is widely distributed in rivers and reservoirs across South Korea, particularly in slow-flowing waters [48]. Its extracts have radical scavenging properties and inhibitory effects on cyanobacterial growth [49]. Moreover, recent studies report additional physiological effects, such as anti-inflammatory activity [50]. The synthesis of its active secondary metabolites is strongly influenced by genetic and environmental factors [51,52,53,54,55,56,57]. While the genetic variation of *M. spicatum* has been analyzed in several countries [58,59,60,61,62,63,64], population-level genetic data for this species remain scarce in South Korea. Thus, considering its ecological importance [50] and biochemical potential [51], examining its genetic variation under different environmental conditions is essential.

In this study, we developed microsatellite markers to investigate the genetic variation and structure of *M. spicatum* populations inhabiting two contrasting habitat types: streams (lotic) and reservoirs (lentic) systems. Our work aims to increase our understanding of how hydrological conditions influence the genetic architecture of submerged macrophytes and to inform future strategies for the sustainable management of native aquatic plant resources.

## 2. Results

### 2.1. Variation in Microsatellite Loci

A total of 30 microsatellite loci were successfully amplified and analyzed across all sampled individuals (Table 1). The polymorphism information content (PIC) values ranged from 0.141 to 0.828, with a mean of 0.530, indicating generally high levels of polymorphism. Four loci (MyspMS19, MyspMS26, MyspMS28, and MyspMS56) exhibited PIC values below 0.3. Across all loci, 148 alleles were detected, ranging from 2 to 9 per locus, with a mean of 4.9. Unique and rare alleles were identified in 25 loci, each containing 1–5 such alleles; the total number of unique and rare alleles was 62. The mean observed heterozygosity (Ho) was 0.443, whereas the mean expected heterozygosity (He) was 0.577. This indicates that observed heterozygosity was generally lower than expected. Despite this, the mean inbreeding coefficient (F_IS_) was −0.566, suggesting an overall excess of heterozygotes. Additionally, six loci exhibited extreme F_IS_ values ranging from −1 to 1, reflecting variation in inbreeding patterns or potential effects of population structure. Genetic differentiation among loci was high, with a mean F-statistic (F_ST_) of 0.556. Locus-specific F_ST_ values ranged from 0.231 to 0.986, indicating considerable variability in genetic differentiation among loci.

### 2.2. Genetic and Clonal Diversity

**Figure 1:**
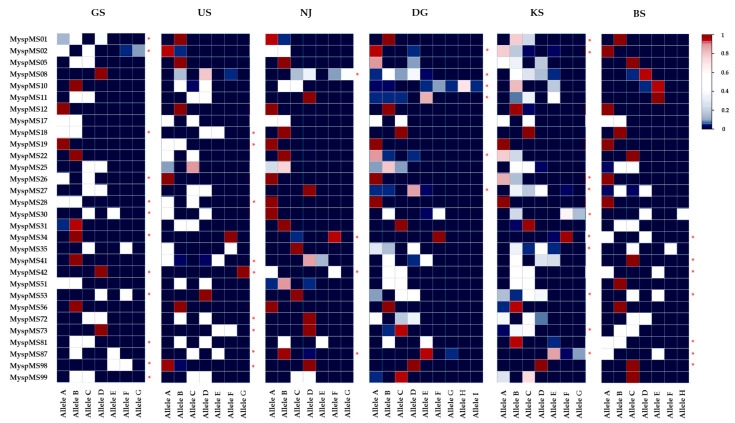
Heatmap of common and unique allele frequency distributions across six *Myriophyllum spicatum* populations (two streams: GS and US, and four reservoirs: NJ, DG, KS, and BS) based on 30 microsatellite loci. Each cell color represents the relative frequency of a particular allele (blue = 0, white ≈ 0.5, red = 1). * indicates loci containing population-specific unique (rare < 0.05) alleles.

Genetic and clonal diversity were analyzed across six populations using 30 microsatellite loci. Among the 148 alleles detected, 62 were unique and rare. The GS population exhibited the highest number of unique alleles, with 15 alleles found across 12 loci. In contrast, the NJ population had only four unique alleles, each from a different locus. Additionally, the KS population had the highest number of rare alleles, totaling seven, whereas no rare alleles (<0.05) were observed in the GS population. However, the NJ population showed the highest number of fixed (1.000) alleles, in 17 loci, whereas the KS population had the fewest, with only 4 fixed loci (Figure 1).

The percentage of polymorphic loci (P_0.95_) ranged from 40.0% in the NJ population to 76.7% in the KS population, with a mean of 61.7% (Table 2). The effective number of alleles per locus (Ae/L) varied from 1.32 (NJ) to 1.75 (KS), with an average of 1.56. Observed heterozygosity (Ho) ranged from 0.220 (NJ) to 0.618 (GS), with a mean of 0.443, whereas expected heterozygosity (He) ranged from 0.157 (NJ) to 0.348 (KS), with a mean of 0.272. These results indicate that the expected heterozygosity was generally lower than observed across populations.

A total of 67 distinct multilocus genotypes (MLGs) were identified from 120 individuals across all populations. The number of genets per population ranged from 4 to 20, with an average of 11.2, and each genotype included a mean of 2.8 ramets (Figure S1). The ratio of genets to sampled individuals (G/N) ranged from 0.200 in the BS population to 1.000 in the KS population. The number of ramets per genotype varied from 2 to 17. Simpson’s diversity index (1–D) ranged from 0.284 (BS) to 1.000 (KS), with a mean value of 0.752. Genotypic evenness (E) ranged from 0.379 (BS) to 1.000 (KS), with an average of 0.818.

**Figure 2:**
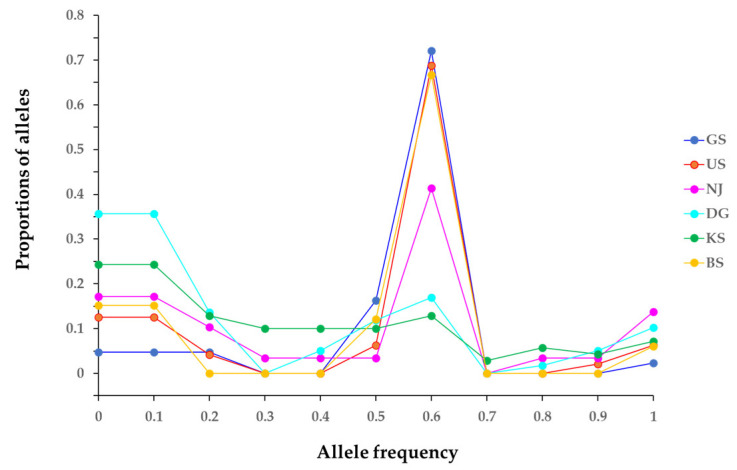
Deviation from an L-shaped allele frequency distribution as evidence of a genetic bottleneck. The analysis is based on 30 microsatellite loci in six populations of *M. spicatum*.

Furthermore, at the species level, expected heterozygosity (He) was higher than the observed heterozygosity (Ho), indicating the possibility of a genetic bottleneck occurring in some populations. The F_IS_ of each population showed a phenomenon of heterozygosity excess. The bottleneck test from the infinite allele model (IAM), stepwise mutation model (SMM), and two-phase model (TPM) revealed strong, significant results for the GS, US, and BS populations—caused by the bottleneck phenomenon (Table 3). Furthermore, although the IAM model suggested a potential bottleneck in the NJ and KS populations, it was not statistically significant in the other models. These results indicate a mode-shift in allele frequency distributions (Figure 2). Notably, several populations exhibited a reversed L-shaped distribution pattern, which is consistent with signatures of a genetic bottleneck.

### 2.3. Genetic Differentiation and Gene Flow

Analysis of 30 microsatellite loci revealed that F_IS_ values were predominantly negative across loci, indicating a general excess of heterozygotes among populations (Table 4). Overall locus-level F_IS_ values ranged from −1.000 to 1.000 (Table 1), whereas within-population F_IS_ values varied between −0.200 and −0.911 (Table 3), reflecting variation in the reproductive mode or clonal propagation.

Pairwise F_ST_ values ranged from 0.161 (between KS and DG) to 0.694 (between BS and NJ), with corresponding Nm estimates ranging from 0.110 to 1.300, respectively (Table 5). The lowest genetic differentiation and the highest estimated gene flow were observed between the geographically proximate KS and DG populations, whereas the greatest differentiation occurred between BS and US.

**Figure 3:**
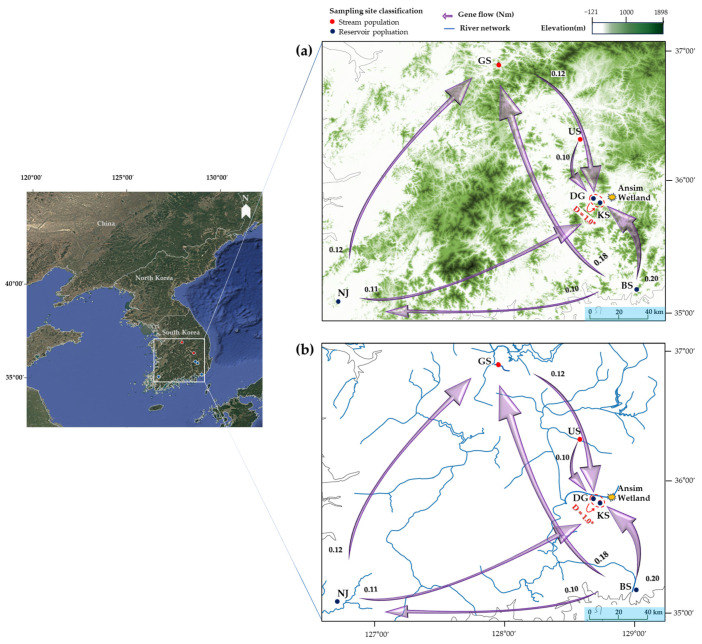
Geographic barriers and directional gene flow of six *Myriophyllum spicatum* populations in South Korea: Elevation (**a**) and River network (**b**) maps. Jost’s D; asterisks (*) indicate significance at *p* < 0.05.

To examine the directionality of gene flow among the six sampling sites, we conducted a directional migration analysis (Figure 3). Most relative migration estimates were low (Nm < 0.2), and although some pairwise directions showed statistically significant values (e.g., BS → KS, NJ → KS), the magnitude of gene flow remained weak, indicating limited genetic connectivity among populations. Despite all six populations being located in hydrologically unconnected and independent waterbodies, a notably strong and statistically significant unidirectional gene flow was detected from DG to KS (Jost’s D = 1.00, *p* < 0.05). A Mantel test examining the relationship between genetic and geographic distances among the six populations revealed no significant correlation (r = 0.420, *p* > 0.05).

### 2.4. Spatial and Genetic Structure

**Figure 4:**
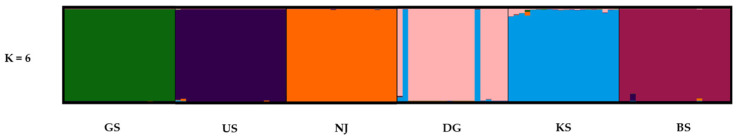
Classification of individuals from six populations of *M. spicatum* into several assumed clusters (ΔK = 6) identified using the STRUCTURE method.

Bayesian clustering analysis implemented in STRUCTURE identified six genetically distinct clusters (K = 6) among the populations, as determined by the ΔK method (Figure 4). Increasing values of K resulted in clearer resolution of the genetic subdivision, with each population corresponding to a discrete genetic cluster, indicating complete genetic separation among them.

**Figure 5:**
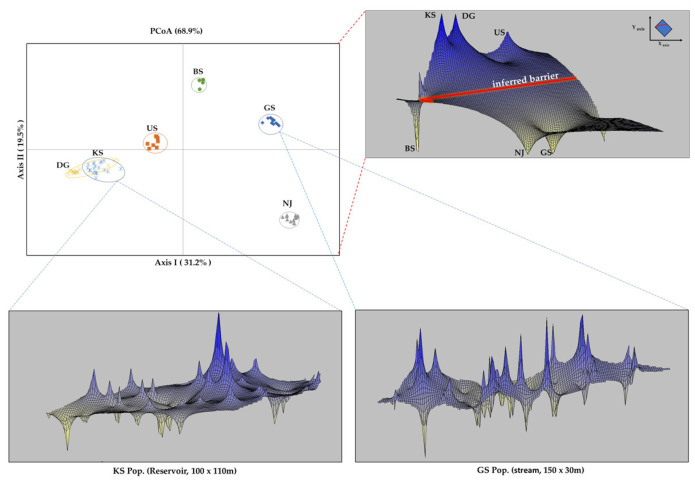
Comparison of spatial genetic structure among six populations of *M. spicatum* using principal coordinate analysis (PCoA) and interpolated genetic landscapes. Bottom panels show surfaces for the KS (reservoir) and GS (stream) populations, based on actual sampling area and pairwise genetic distances, illustrating spatial genetic structure.

Principal coordinate analysis (PCoA) based on MLG data accounted for 68.9% of the total genetic variation across the three axes (Figure 5). Axis I, which explained 31.2% of the variation, separated the US, DG, and KS populations from the GS, NJ, and BS populations. The DG and KS populations were positioned proximally, whereas NJ exhibited strong divergence relative to the other populations.

**Figure 6:**
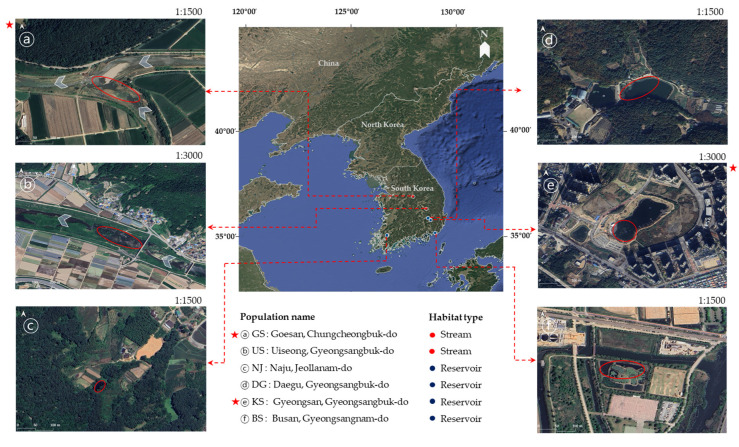
Geographic distribution of six *M. spicatum* sampling sites in South Korea. The map illustrates the national distribution of stream (red) and reservoir (blue) populations, while panels (ⓐ–ⓕ) show high-resolution satellite images of the sampling habitats (scale 1:1500 and 1:3000). Sampling sites marked with red asterisks (★) were included in spatial genetic structure analyses.

Spatial genetic structure analyses identified a pronounced genetic discontinuity between the KS–DG–US and NJ–GS clusters, suggesting the presence of an inferred genetic barrier. The BS population was genetically differentiated from all other populations and formed an isolated unit in both ordination and clustering analyses. The analyzed spatial genetic patterns in two ecologically distinct populations—GS (stream) and KS (reservoir)—exhibited the most contrasting spatial structures. In the KS population (reservoir), intra-population genetic similarity was highest near the reservoir center and declined progressively toward the margins. Spatial autocorrelation was significantly positive within 20 m, with genetic similarity persisting up to a 60 m distance. Conversely, the GS population (stream) displayed a directional spatial structure that may reflect downstream clonal spread (R → L), as supported by the spatial autocorrelation analyses and field observations (Figure 6). Positive spatial autocorrelation was observed within 15 m and remained detectable up to 140 m.

## 3. Discussion

### 3.1. Variation in Microsatellite Loci

Microsatellite markers are powerful tools for detecting genetic variations and identifying diverse alleles, making them essential for population genetic studies [65,66]. In this study, the selected loci exhibited a wide range of PIC, from 0.141 to 0.828 (Table 2), reflecting their capacities to reveal genetic diversity. For robust analysis, we prioritized markers with PIC values above 0.5 to reduce potential bias caused by excessive allele variability. Furthermore, loci with PIC values below 0.3 were also retained based on comprehensive consideration of the clonal reproductive strategy of species and supporting evidence from previous studies [67,68]. Although these loci exhibited limited polymorphism, they harbored private alleles unique to specific populations, providing valuable insights into population differentiation and regional specificity (Figure 1).

The observed mean F_IS_ value (−0.566) and excessive heterozygosity suggest that many loci deviated from the Hardy–Weinberg equilibrium toward heterozygote excess, likely due to clonal reproduction or outcrossing. Extremely high F_IS_ values (1.000) in specific populations support clonal propagation. The broad range of population-level F_IS_ values (−1.000 to 1.000) indicates variations in reproductive strategies across sites, with evidence of localized inbreeding. These results reflect the species’ mixed reproductive system and the influence of habitat conditions on genetic structure. This pattern and high F_ST_ values (mean F_ST_ = 0.556) indicate strong genetic differentiation among populations. Therefore, the microsatellite markers developed in this study are effective for detecting population structure and inferring the genetic origin of individuals.

Considering that *M. spicatum* is an aquatic species widely distributed worldwide, its gene flow is likely more limited than that of terrestrial plants. In species such as *M. spicatum* with sexual and clonal reproduction, the reproductive mode and environmental disturbance can significantly shape population structure. Hence, instead of relying solely on PIC values or heterozygosity levels, we selected markers through an integrated approach that considered allele frequency distributions and population-specific dynamics, and the validity of the developed microsatellite markers was confirmed. The subsequent sections provide a detailed discussion of the genetic analysis outcomes.

### 3.2. Genetic and Clonal Diversity

We analyzed the genetic diversity and clonal propagation of six *M. spicatum* populations using 30 microsatellite loci (Table 2). In our study, the species-level genetic diversity across all loci (expected heterozygosity, He = 0.577) was substantially lower than that reported by Cao et al. [62] (total He = 0.973) and Wu et al. [60] (Nei’s genotypic diversity [He], Overall D = 0.995).

Our results may reflect variations in environmental factors such as habitat area, population size, natural selection pressure, and disturbance intensity, as well as variations in the specificity of the developed markers. These findings suggest that the South Korean populations examined in this study may experience more constrained genetic variation than those reported in previous studies. Although the number of study sites was limited, the observed patterns were generally consistent with those reported in previous research.

Aquatic plants primarily propagate clonally, and many exhibit high clonal diversity [69], which can accumulate through occasional seed recruitment or be maintained through clonal growth in relatively stable environments [70]. Such patterns are often associated with habitats such as ponds, lakes, or areas with minimal water flow [71,72]. *M. spicatum* is an anemophilous species [73]; however, it may fail to produce seeds in flowing waters [74]. Thus, under high-flow conditions, sexual reproduction can be limited [71].

In our study, genetic and clonal diversity differed markedly between stream (GS and US) and reservoir (NJ, DG, KS, and BS) populations (Table 2). In stream habitats, allelic richness and expected heterozygosity were relatively low, and both GS and US were dominated by a single MLG. This MLG showed a downstream-oriented spatial signal, consistent with clonal spread (Figure S2). These results align with patterns previously reported [31,61]. Such a pattern may be associated with hydrological infrastructure (e.g., dams, sluice gates, and other water control structures), which could influence the balance between sexual and clonal reproduction.

Both GS and US are in watersheds subject to recurrent summer monsoon flooding and flow regulation. According to the national government-operated water quality monitoring [75] and flood forecasting systems in South Korea [76], the sites where the two stream populations are located are classified as flood-prone areas with existing flow regulation infrastructure. In South Korea, the monsoon and typhoon season typically occurs between June and September [77], which often overlaps with the reproductive period of *M. spicatum* [33]. Extreme rainfall events during this time could, therefore, substantially disrupt reproduction. Additionally, nearby agricultural activities and site management practices may further alter local hydrological and ecological conditions [76,77]. The clonal structure of GS and US, combined with heterozygosity excess under IAM, SMM, and TPM (Table 3), is consistent with recent bottleneck events potentially associated with environmental disturbances, as strong significance was detected across all three bottleneck detection models.

Water quality monitoring data for the two stream populations (March to September, 2016–2023; Table S3) indicate that mean total phosphorus and total nitrogen concentrations were within commonly eutrophic ranges [75], reaffirming the strong capacity of *M. spicatum* to survive, establish, and reproduce even under flow-affected river conditions [78].

On the contrary, the KS population showed the highest clonal diversity (G/N = 1.00), with every sampled individual having a unique MLG. This value exceeds the clonal diversity reported by Cao et al. [62] (mean G/N = 0.62). The discrepancy may be attributable to differences in site scale and sampling methodology: we analyzed a single population within one site, using a minimum spacing of 5 m between samples, whereas Cao et al. sampled multiple subpopulations within a single lake at approximately 1 m intervals. Consequently, our approach may have detected a broader range of diverse MLGs.

Two hypotheses might explain this pattern: First, high population density in a stable environment may have promoted competition, suppressing clonal growth and favoring sexual reproduction [79,80]. The large, deep reservoir habitat may also enable size variation that enhances individual persistence under low-resource conditions or limited light availability [81,82]. Second, the observed diversity may reflect the long-term accumulation and persistence of MLGs maintained by clonal propagation and occasional sexual recruitment [15,65,83]. However, the KS population, located in a long-standing reservoir, experienced anthropogenic modifications between 2014 and 2022, owing to lake park development. Satellite imagery of the reservoir revealed that the entire water body was drained as a result of construction activities (imagery date: September 2015 to April 2016; Figure S2), suggesting that the pre-development population size may have been larger than it is at present. If the lake bed has not been fully restructured, a broader range of genotypes—undetected in our sampling—could remain in deeper sediment layers.

By contrast, the BS population, which showed the lowest clonal diversity (G/N = 0.20), was located in a manmade pond established near a river in 2010. The initial establishment of *M. spicatum* may have resulted from intentional planting or recolonization from nearby sources (imagery date: September 2009 to April 2016; Figure S2). However, continued human interventions—such as plant removal and hydrological management—appear to have favored the selective persistence of a few stress-tolerant MLGs. BS exhibited the lowest clonal diversity and the strong dominance of a few clones. Among the four detected ramets, one MLG accounted for more than 60% of individuals (Figure S1). In disturbed environments, specific MLGs may prevail, owing to their greater adaptability [84,85,86,87,88,89,90,91,92]. Nonetheless, if only a small number of individuals retain their sexual capacity, long-term fitness could be compromised by processes such as Muller’s ratchet [93,94,95].

The contrasting results of the KS and BS populations, despite both originating from the same habitat type, suggest that the divergent patterns observed between stream and reservoir populations are driven by regional-scale ecological and anthropogenic processes rather than by solely site-specific factors. This highlights the need for broader-scale surveys in future research.

### 3.3. Genetic Differentiation and Gene Flow

Building on these differences in genetic and clonal diversity, we next examined the extent of genetic differentiation and patterns of gene flow among the six *M. spicatum* populations. High genetic differentiation in this species has previously been linked with clonal reproduction and may be further reinforced by geographic isolation, which limits gene flow [23,59,96]. Except in cases where populations were sampled as subpopulations within the same watershed (Φ_ST_ = 10% [62]), among-population differentiation is generally high in *M. spicatum*, as reported by Wu et al. (Φ_PT_ = 0.521) [60] and Thum et al. (Φ_ST_ = 58%) [63]. Consistent with these findings, our analysis revealed high variations within populations and genetic differentiation among populations, with 57% (F_ST_ = 0.569) of the total genetic variance explained among populations.

Wu et al. [60] highlighted the role of geographic barriers, together with climatic conditions, on shaping genetic differentiation. Consistent with this, although directional gene flow was significant in a few pairwise comparisons, estimated Nm values were consistently low (all < 0.2), indicating that gene flow among populations was limited, despite the directional signal (Figure 3). Notably, despite the much smaller spatial extent of our sampling area, we observed a similar pattern, indicating that geographic barriers can emerge even across relatively narrow areas.

Genetic differentiation between the geographically unconnected DG and KS populations was the lowest (F_ST_ = 0.161, Nm = 1.300), suggesting the possibility of ongoing gene flow between them (Table 5). This pattern may reflect ecological connectivity through natural gene dispersal processes and anthropogenic introduction pathways associated with reservoir management of aquatic vegetation. Aquatic macrophytes are often transplanted for purposes such as water purification or landscape enhancement; however, mechanical removal activities may inadvertently disperse vegetative fragments to new locations.

The trajectory of seed dispersal can provide insights into the vector species, their life histories, and the timing of ingestion and excretion [97]. *M. spicatum* seeds are consumed and dispersed by various waterbirds [60,98,99], and highly mobile vectors such as birds and fish can transport seeds across hundreds of kilometers [100,101]. In this context, we explored ecological factors: for DG and KS, the proximity of both populations to the Ansim Wetland, a migratory bird sanctuary in the Geumho River basin (linear distance, <8 km) [102], raises the possibility of opportunistic gene flow by bird-mediated dispersal (Figure 3).

Despite the potential for biotic dispersal, the limited extent of gene flow observed in this study may suggest that *M. spicatum* has relatively low palatability or ecological value as a food source for birds and fish [35,46,58,103]. When gene flow or genetic differentiation is confined to localized areas, unique alleles within each population may contribute to the observed genetic structuring, as further supported by the PCoA and STRUCTURE analysis results (Figure 4 and Figure 5). These findings imply that gene flow in *M. spicatum* primarily occurs over short distances, with local environmental selection pressures further reinforcing genetic differentiation. Consequently, the genetic connectivity observed between DG and KS populations cannot be fully explained by natural dispersal mechanisms alone and may involve indirect anthropogenic influences.

### 3.4. Spatial and Genetic Structure

The pronounced genetic differentiation observed among six populations of *M. spicatum* implies limited gene flow [59,63,64,104,105]. Genetic barriers were observed between the NJ–GS and KS–DG–US population groups, while the BS population consistently showed patterns of genetic isolation even in PCoA (Figure 5). Because the studied populations are not hydrologically or geographically connected, the observed genetic patterns are likely shaped by localized environmental factors and restricted dispersal.

Spatial genetic structure analyses further revealed contrasting patterns between KS (reservoir) and GS (stream). KS exhibited a gradual genetic gradient, whereas GS showed directional clonal spread aligned with downstream flow (Figure 5 and Figure 6). These contrasts are broadly consistent with the results of previous studies on spatial genetic structure and dispersal in aquatic macrophytes [23,106,107,108,109,110], supporting the idea that physical habitat characteristics strongly influence local genetic structure. Collectively, our findings indicate that the spatial genetic structure of *M. spicatum* in South Korea results from a complex interplay of reproductive mode, habitat-specific conditions, and anthropogenic disturbances.

Although this study contrasted populations by habitat type, populations distributed along a continuous watershed may display different, connectivity-driven gene flow regimes. Moreover, environmentally mediated shifts in reproductive mode may lead to population-specific differences in seed germination and establishment. Together, these factors could generate diverse patterns of genetic diversity and population structure. Future research should integrate population genetic analyses with ecological and physiological assessments linked to life history and reproductive strategies.

### 3.5. Implications for Conservation and Sustainable Management

Long-term monitoring of the origin of collected individuals and historical habitat changes is essential to improve the efficiency of future conservation and resource management [111]. Populations experiencing substantial environmental fluctuations, such as stream-type habitats, may serve as important areas for the utilization of specific genotypes and for ecological monitoring. In South Korea, for example, stream populations such as GS and US and the reservoir populations NJ and BS illustrate this pattern, whereas reservoir population KS maintained comparatively rich genotypic diversity, despite a history of anthropogenic modification, indicating their potential value as key resources for the conservation of *M. spicatum* diversity.

This contrast is consistent with that in previous studies, indicating that clonal reproduction can lead to different outcomes in population structure and genetic diversity depending on habitat conditions [8]. Particularly, You et al. [112] demonstrated that continuous environmental fluctuations within habitats can promote and reinforce the dominance of specific clones within populations. Although not all environmental factors can be controlled, the presence or absence of planned watershed management may either support the conservation of aquatic plant resources or exert negative impacts. Incorporating such habitat-specific genetic structures into aquatic plant management could contribute to resource conservation and effective management strategies not only in South Korea but also in other regions facing similar hydrological gradients [113].

## 4. Materials and Methods

### 4.1. Sample Preparation

For the genetic analysis, a total of 120 individuals of *M. spicatum* were collected from six populations in 2023 (Figure 6 and Table S2). Each population was represented by 20 individuals, selected to reflect variation in habitat types and disturbance regimes. The stream populations (GS and US) are classified as local rivers managed by municipal governments. Site-specific information on hydrological management and usage was compiled through field observations, historical changes traced via satellite imagery, and documentation provided by the respective local authorities [76,77,114]. All individuals sampled within each population were separated by at least 5 m to avoid collecting the same clone. Prior to sampling, the taxonomic identity of *M. spicatum* was confirmed by a professional plant taxonomist through cross-validation.

The six sampling sites were strategically selected to reflect spatial and ecological heterogeneity, including differences in habitat types (e.g., streams and reservoirs) and disturbance intensity (Figure 6). To assess potential sources and patterns of habitat disturbance, satellite imagery was obtained for each site and its surrounding landscape. These images were extracted from Google Satellite basemaps and visualized using QGIS version 3.34 (accessed June 2025).

To examine the formation of spatial genetic structure within populations, we selected the GS and KS populations, which exhibited relatively intact clonal patches with visible spatial distribution patterns. Twenty individuals were sampled from each site within defined areas of 150 × 30 m and 100 × 110 m for GS and KS populations, respectively. A minimum distance of 5 m was maintained between individuals to reduce the likelihood of resampling identical genets. The geographic location of each sampled individual was georeferenced, and these coordinates were used for subsequent spatial genetic analyses. When the geographic coordinates of sampled individuals could not be recorded directly in the field, high-resolution satellite imagery was used to match the sampling locations with field photographs and secure accurate spatial coordinates.

### 4.2. Microsatellite Marker Development and Polymerase Chain Reaction (PCR) Amplification

Microsatellite regions were identified using a genome assembly and screened using the Microsatellite (MISA) tool (https://webblast.ipk-gatersleben.de/misa/, accessed on 23 August 2023). Screening parameters were set to detect a minimum of 10 repeats for di-nucleotide motifs, and at least 4 repeats for tri-, tetra-, penta-, and hexa-nucleotide motifs. Primers were designed using Primer3 (https://github.com/primer3-org/primer3, accessed on 23 August 2023) with the following criteria: an amplicon size of 100–300 bp, primer length of 20–24 bp, GC content between 40 and 60%, and melting temperature of 58 °C. Primer specificity was confirmed using SnapGene (GSL Biotech, Chicago, IL, USA) to ensure that each primer pair uniquely bound to the target sequence.

A total of 100 primer pairs were initially designed. PCR conditions were optimized, and each primer was tested on 6 individuals per population to assess polymorphism. Thirty polymorphic and reproducibly amplified loci were selected for further analysis of the total samples (Table S1). The final set of 30 validated microsatellite loci was submitted to GenBank, and accession numbers (PV231646–PV231675) are provided.

Microsatellite loci amplification was performed using the Mastercycler^®^ Pro Gene Amplifier (Eppendorf, Hamburg, Germany). PCR was conducted at a total volume of 20 μL, using H-Star Taq DNA Polymerase (Biofact, Daejeon, Republic of Korea). Fluorescent labeling was performed following the protocol described by Schuelke [115], using four fluorescent dyes (6-FAM, VIC, NED, PET). Each reaction mixture contained a microsatellite-specific forward primer (0.4 μM), a reverse primer synthesized with an M13 tail sequence (5′-TGTAAAACGACGGCCAGT, 0.8 μM), and a fluorescently labeled M13 primer (0.4 μM). The PCR cycling conditions consisted of initial denaturation at 94 °C for 5 min, followed by 30 cycles of denaturation at 94 °C for 30 s, annealing at 58 °C for 45 s, and extension at 72 °C for 45 s. This was followed by 12 cycles at 94 °C for 30 s, 53 °C for 45 s, and 72 °C for 45 s. A final extension step was performed at 72 °C for 10 min. PCR products were mixed with GeneScan™ 500 ROX Size Standard Ladder (Applied Biosystems, Foster City, CA, USA) and HiDi™ formamide, denatured at 95 °C for 2 min, and then cooled to 4 °C. Allele sizing was performed using an ABI 3730xl DNA Analyzer (Applied Biosystems), and genotyping was conducted using the GeneMarker^®^ software ver. 2.6.7 (SoftGenetics, State College, PA, USA). To visualize the distribution of common and population-specific alleles across 30 microsatellite loci, a heatmap was generated using the Heatmapper web tool (http://www.heatmapper.ca/expression/ accessed on 7 May 2025), based on allele frequency data from each sampling site.

### 4.3. Genetic and Clonal Diversity Analysis

The MICROCHECKER software ver. 2.2.3 [116] was used to examine the presence or absence of scoring errors in the microsatellite loci. The genetic analyses were performed based on the frequency of the aforementioned marker bands, following the categorization method of Steeves et al. [117]. PIC values were estimated using Cervus ver. 3.0.7 [118], whereas F-statistics were derived from Nei’s method implemented in FSTAT ver. 2.9.4 [119].

Genetic diversity was analyzed in the percentage of polymorphic loci (P_0.95_), the mean number of alleles per locus (A), the effective number of alleles per locus (Ae), and the observed (Ho) and expected (He) heterozygosity, using the Popgene software v1.32 [120]. Clonal diversity was analyzed by counting the number of ramets and genotypes. The ratio of different MLGs (G/N) to the total number of sampled individuals was used to estimate genotypic diversity. Additionally, the richness index (D), Simpson’s unbiased diversity index (1 − D), and genotypic evenness (E) were manually calculated based on MLG frequencies, following Simpson (1949) [121] and Pielou (1969) [122]. Wilcoxon’s signed-rank test was conducted using the BOTTLENECK software ver. 1.2.02 [123], under the assumptions of the IAM, SMM, and TPM, to identify populations that had recently experienced a decline in effective size. The genetic bottleneck test was conducted by comparing one- and two-tailed tests to determine significance and minimize the risk of distorted results. Significance was assessed using sign tests and Wilcoxon one-tailed tests, with estimation based on 1000 replications. Mode-shift indicator graphs were also generated to visualize allele frequency distributions across populations.

### 4.4. Genetic Differentiation and Gene Flow

To determine the genetic differentiation in each population, Wright’s F-statistics (F_IS_, F_IT_, F_ST_; [124]) were calculated using FSTAT ver. 2.9.4 [119]. F_IS_ values were estimated for polymorphic loci, applying the 0.95 criterion, and their significance was tested through permutation tests with 180,000 randomizations. To assess population differentiation, the statistical significance of F_ST_ and F_IT_ was evaluated by calculating the 95% confidence intervals, and gene flow (expected as Nm) was estimated from the F_ST_ value using GenAIEx ver. 6.503 [125]. To examine the correlation between genetic differentiation and geographic distance among populations, Mantel tests [126] were conducted using the IBD software (isolation by distance) ver. 1.52 [127]. To assess directional gene flow and detect asymmetric migration among populations, we employed the DivMigrate online application (https://popgen.shinyapps.io/DivMigrate-online/ accessed on 7 May 2025), developed as part of the diveRsity R package ver. 1.9.90 [128]. The analysis was based on Gst estimates, with 1000 bootstrap replicates used to test the significance of asymmetric migration between population pairs. Additionally, Jost’s D was calculated as an alternative measure of genetic flow.

### 4.5. Spatial and Genetic Structure

To investigate the genetic structure of each population, we employed STRUCTURE ver. 2.3.4 [129], which identified discrete genetic clusters and estimated the proportion of individual genotypes assigned to each cluster. Simulations were run 10 times for each ΔK value (1–10), with 1,000,000 MCMC iterations following a burn-in of 100,000, using the admixture model and assuming correlated allele frequencies [130,131]. The optimal K value was visualized using the Structure Selector web tool [132].

Pairwise genetic distances among individuals were calculated, and Principal Coordinate Analysis (PCoA) was performed using GenAlEx ver. 6.503 [125] to generate relative spatial coordinates. The spatial positions of sampled individuals and the distributional extent of each population were standardized using the vegan package ver. 2.6.4 [133] in R ver. 4.3.2 [134]. These coordinates were subsequently scaled to reflect the actual sampling area accurately. The scaled coordinates and the genetic distance matrix were used in the Alleles in Space (AIS) software ver. 1.0 [135] to infer and visualize fine-scale spatial genetic structure. For the overall analysis across six populations, the XY coordinate space was divided into a 150 × 150 grid with a uniform weighting of 1. To assess within-population spatial genetic structure, grid settings were adjusted to match the actual sampling area for each habitat: a 100 × 110 grid for the KS population (reservoir) and a 150 × 30 grid for the GS population (stream).

## 5. Conclusions

Although *M. spicatum* is globally distributed, its ecological traits and genetic composition vary across regions in response to environmental heterogeneity, as shown by genetic studies from temperate regions of China, North America, and Europe. In this study, our analysis of six South Korean populations revealed a more nuanced and complex genetic structure.

The results further confirm that reproductive strategies of *M. spicatum* populations shift with environmental variability and highlight the value of long-term monitoring for understanding habitat-specific genetic structures. The balance between sexual reproduction and clonal propagation differed among habitats, offering evolutionary evidence of the species’ strong persistence and establishment capacities. In more variable environments, clonal propagation is critical for population survival, whereas in more stable habitats, higher rates of sexual reproduction likely support genetic diversity. Such dynamics may contribute to a more restricted spatial genetic structure than previously anticipated.

In South Korea, considerable resources are allocated annually to water quality management [111,136]. However, climate-driven changes such as unseasonal algal blooms are exerting increasing pressure on freshwater ecosystems [137]. Our findings underscore the importance of national initiatives aimed at mitigating environmental degradation and promoting ecosystem restoration. Although *M. spicatum* has been linked to ecological challenges in specific regions, its functional role in aquatic systems may ultimately depend on local management strategies.

The 30 microsatellite markers developed and applied in this study demonstrated strong discriminatory power among populations, underscoring their utility for long-term genetic monitoring and comparative studies in other regions. By providing novel insights into the genetic and clonal characteristics of *M. spicatum* populations in South Korea, recently re-evaluated in terms of their resource value, we hope this work will serve as a valuable resource for future research and management strategies.

## Figures and Tables

**Table 1:** Assessment of genetic variations of 30 microsatellite loci developed from *M. spicatum.* PIC: polymorphism information content; A: number of alleles; Ae/L: effective number of alleles, for all loci; Unique allele: alleles with frequency >0.05 were classified as common and those <0.05 as rare; Ho: observed heterozygosity; He: expected heterozygosity; F_IS_: inbreeding coefficient; F_ST_: genetic differentiation.

GeneBank Accession No.	Locus	PIC	A	Ae/L	Unique Allele		Ho	He	F_IS_	F_ST_
					Common	Rare				
PV231646	MyspMS01	0.409	4	1.96	2	0	0.250	0.449	−0.418	0.650
PV231647	MyspMS02	0.367	7	1.88	3	1	0.433	0.392	−0.526	0.314
PV231648	MyspMS05	0.592	4	2.75	0	0	0.225	0.652	−0.021	0.702
PV231649	MyspMS08	0.548	7	3.73	2	0	0.292	0.588	0.289	0.341
PV231650	MyspMS10	0.673	9	2.77	4	1	0.392	0.708	−0.213	0.589
PV231651	MyspMS11	0.658	5	3.66	1	0	0.492	0.709	−0.613	0.614
PV231652	MyspMS12	0.381	3	1.96	0	1	0.008	0.504	0	0.986
PV231653	MyspMS17	0.555	3	2.63	0	0	1.000	0.625	−1	0.231
PV231654	MyspMS18	0.644	5	2.90	3	0	0.333	0.694	−1	0.792
PV231655	MyspMS19	0.141	2	1.13	1	0	0.167	0.153	−1	0.500
PV231656	MyspMS22	0.590	4	2.57	1	0	0.258	0.663	−0.480	0.770
PV231657	MyspMS25	0.569	4	3.02	0	0	0.492	0.640	−0.184	0.394
PV231658	MyspMS26	0.185	3	1.30	2	0	0.217	0.198	−0.709	0.401
PV231659	MyspMS27	0.486	6	2.25	0	2	0.658	0.538	−0.617	0.278
PV231660	MyspMS28	0.272	3	1.34	2	0	0.333	0.292	−1	0.474
PV231661	MyspMS30	0.820	8	5.57	3	0	0.725	0.840	−0.686	0.534
PV231662	MyspMS31	0.387	3	2.02	1	0	0.342	0.508	−0.783	0.665
PV231663	MyspMS34	0.494	6	1.67	4	1	0.192	0.530	−0.776	0.825
PV231664	MyspMS35	0.725	6	4.46	1	0	0.700	0.763	−0.565	0.459
PV231665	MyspMS41	0.728	5	3.46	2	0	0.492	0.766	−0.499	0.616
PV231666	MyspMS42	0.828	7	5.60	4	0	0.625	0.847	−0.87	0.648
PV231667	MyspMS51	0.484	4	2.29	0	0	0.625	0.521	−0.713	0.339
PV231668	MyspMS53	0.617	6	2.97	3	0	0.600	0.676	−0.593	0.488
PV231669	MyspMS56	0.247	2	1.46	0	0	0.000	0.289	1	0.951
PV231670	MyspMS72	0.592	4	3.00	1	0	0.775	0.648	−0.736	0.351
PV231671	MyspMS73	0.691	6	3.46	2	1	0.483	0.735	−0.811	0.678
PV231672	MyspMS81	0.577	5	2.15	3	0	0.833	0.611	−0.927	0.332
PV231673	MyspMS87	0.566	7	2.29	2	2	0.558	0.614	−0.785	0.536
PV231674	MyspMS98	0.645	6	2.17	4	1	0.175	0.682	−0.909	0.885
PV231675	MyspMS99	0.441	4	2.04	1	0	0.608	0.478	−0.823	0.342
	Mean	0.530	4.9	2.68	1.73	0.33	0.443	0.577	−0.566	0.556
	Overall	0.554	148	80.46	52	10	0.443	0.577	−0.610	0.569

**Table 2:** Genetic and clonal diversity in six populations of *M. spicatum* analyzed by 30 microsatellite loci. N: number of samples; P _(0.95)_: percentage of polymorphic loci; Ae/L: effective number of alleles per locus; Ho: observed heterozygosity, He: expected heterozygosity, G_(R)_: number of multilocus genotypes (MLGs) per population (range of genets that have a ramet); G/N: ratio of genotype; D: Simpson’s diversity index (1–D); E: evenness of genotype.

Population	N	Genetic					Clonal			
		P _(0.95)_	A	Ae/L	Ho	He	G_(R)_	G/N	D	E
GS	20	66.7	53	1.67	0.618	0.327	11 (2–9)	0.550	0.763	0.839
US	20	70.0	56	1.64	0.608	0.324	8 (2–12)	0.400	0.567	0.648
NJ	20	40.0	46	1.32	0.220	0.157	12 (2–8)	0.600	0.901	0.982
DG	20	66.7	69	1.52	0.352	0.252	12 (2–8)	0.600	0.948	1.000
KS	20	76.7	74	1.75	0.427	0.348	20 (0)	1.000	1.000	1.000
BS	20	50.0	48	1.45	0.432	0.225	4 (2–17)	0.200	0.284	0.379
Mean	20	61.7	57.7	1.56	0.443	0.272	11.2	0.558	0.752	0.818
Total	120	100	148	2.80	0.443	0.577	67 (53)	0.558	0.959	0.987

**Table 3:** Results of the Wilcoxon signed-rank test for bottleneck detection (one- and two-tailed analyses) under three models: infinite allele model (IAM), two-phase mutation model (TPM), and stepwise mutation model (SMM). F_IS_ = coefficient of inbreeding, calculated from 30 microsatellite loci. Significance levels are denoted as follows: *p* < 0.05 *, *p* < 0.001 ***.

Population	He	IAM	SMM	TPM	F_IS_
GS	0.327	0.250 ***	0.278 ***	0.299 ***	−0.888
US	0.324	0.256 ***	0.285 ***	0.306 ***	−0.870
NJ	0.157	0.261 *	0.289	0.314	−0.382
DG	0.252	0.353	0.396	0.431	−0.372
KS	0.348	0.331 *	0.369	0.406	−0.200
BS	0.225	0.258 ***	0.284 ***	0.308 ***	−0.911

**Table 4:** Fixation index (F_IS_) values for 30 microsatellite loci across six populations of *Myriophyllum spicatum*, along with overall estimates of F_IS_, F_IT_, and F_ST_ based on F-statistics. Significance levels are denoted as follows: *p* < 0.05 *, *p* < 0.01 **, *p* < 0.001 ***.

Locus	GS	US	NJ	DG	KS	BS	
MyspMS01	−0.570 ***	-	−0.027	-	−0.267	-	
MyspMS02	−0.498 **	−0.027 ***	−1.000 ***	−0.027	−0.200	-	
MyspMS05	−0.727 **	-	-	1.000	0.220	-	
MyspMS08	-	0.714	0.861	−0.376 **	−0.016	1.000	
MyspMS10	-	−0.905 ***	−1.000 ***	0.411	0.835	1.000	
MyspMS11	−1.000 ***	−1.000 ***	-	0.645	−0.553 **	-	
MyspMS12	-	-	-	-	0.000	-	
MyspMS17	−1.000 ***	−1.000 ***	−1.000 ***	−1.000 ***	−1.000 ***	−1.000 ***	
MyspMS18	−1.000 ***	−1.000 ***	-	-	-	-	
MyspMS19	-	−1.000 ***	-	-	-	-	
MyspMS22	-	−1.000 ***	-	0.479	−0.267	-	
MyspMS25	−1.000 ***	1.000	1.000	0.719	−0.615 **	−0.905 ***	
MyspMS26	−1.000 ***	-	-	-	−0.152	-	
MyspMS27	−1.000 ***	−1.000 ***	-	0.791	−0.376 *	−0.818 ***	
MyspMS28	−1.000 ***	−1.000 ***	-	-	-	-	
MyspMS30	−1.000 ***	−1.000 ***	-	−0.905 ***	0.370	−1.000 ***	
MyspMS31	1.000	−1.000 ***	-	-	0.000	−1.000 ***	
MyspMS34	-	-	−0.027	-	0.000	−1.000 ***	
MyspMS35	−1.000 ***	−1.000 ***	-	−0.520 ***	0.493	−1.000 ***	
MyspMS41	-	−0.818 ***	0.782	−0.744 ***	−0.473 **	-	
MyspMS42	-	-	−1.000 ***	−1.000 ***	−0.484 *	−1.000 ***	
MyspMS51	−1.000 ***	−1.000 ***	−0.056	−1.000 **	−0.100	-	
MyspMS53	−0.900 ***	-	-	−0.520	−0.122	−1.000 ***	
MyspMS56	-	-	-	-	1.000	-	
MyspMS72	−0.727 **	−1.000 ***	-	−0.510	−0.524 **	−1.000 ***	
MyspMS73	-	−1.000 ***	-	−0.027 ***	−0.587 **	−1.000 ***	
MyspMS81	−1.000 ***	−1.000 ***	−1.000 ***	−0.810	−0.027	−1.000 ***	Overall
MyspMS87	−1.000 ***	−1.000 ***	0.000	−0.027	−0.092	−0.900 ***	Nm = 0.190
MyspMS98	−1.000 ***	0.000	-	-	-	-	F_IS_ = −0.610
MyspMS99	−1.000 ***	−1.000 ***	−1.000 ***	−0.027	−0.357	-	F_IT_ = 0.305
All	−0.888 ***	−0.870 ***	−0.382 ***	−0.372 ***	−0.200 ***	−0.911 ***	F_ST_ = 0.569

**Table 5:** Correlation coefficients between all of the combinations of pairwise differentiation (F_ST_; below) and gene flow (Nm; top). Values based on F_ST_ values.

Pop.	GS	US	NJ	DG	KS	BS
GS	-	0.219	0.167	0.161	0.229	0.189
US	0.533	-	0.134	0.111	0.153	0.110
NJ	0.600	0.652	-	0.229	0.315	0.175
DG	0.608	0.692	0.522	-	1.300	0.169
KS	0.521	0.620	0.442	0.161	-	0.215
BS	0.569	0.694	0.588	0.597	0.538	-

## Data Availability

The original contributions presented in this study are included in the article. Further inquiries can be directed to the corresponding author.

## Supplementary Materials

The following supporting information can be downloaded at: https://www.mdpi.com/article/10.3390/plants14172648/s1, Table S1: Amplification information primer sequences and characteristics of 30 microsatellite loci developed from *Myriophyllum spicatum*; Table S2: Summary of population information for six *M. spicatum* sampling sites in South Korea; Table S3: Annual water quality metrics (March–September means, 2016–2023) for the GS and US sites where *M. spicatum* was sampled; Figure S1: UPGMA tree based on Nei’s genetic distance, calculated for clonal and genetic relationships among six *M. spicatum* populations; Figure S2: Satellite imagery showing land use changes at two *M. spicatum* sampling sites in South Korea.
