# Supplementary material for: Patterns of Genetic and Clonal Diversity in Myriophyllum spicatum in Streams and Reservoirs of Republic of Korea
Source: Plants (Basel). 2025 Aug 26;14(17):2648. doi: 10.3390/plants14172648 (PMC12430124; doi:10.3390/plants14172648)
Supplement: Supplementary file 1 [file plants-14-02648-s001.zip › plants-3734871-supplementary.docx]

**Supplementary Material**

| Locus | Primer sequence (5’-3’) | | | | Allele range (bp) | | Temp. (℃) | Flu. |
| --- | --- | --- | --- | --- | --- | --- | --- | --- |
|  | Forward | Reverse | | |  |  |  |  |
| MyspMS01 | CCCTCTCACTTCGTAGTCGC | AGTAGAGCCAGTGGACCTGT | | | 215-224 | 60 | | 6-FAM |
| MyspMS02 | TGTAGTGCGTGTCGTTGACCTT | | ACAGATTGGCATGCATCCTG | | 220-264 | 60 | | 6-FAM |
| MyspMS05 | TCAGGTTGATCGCGTGATGT | TCGCTCGAAGGTGTTATCGG | | | 220-226 | 60 | | 6-FAM |
| MyspMS08 | TCCACAAGCAAGCAAGCAAC | GCCGTGAAATGTTGCAATGC | | | 221-233 | 60 | | 6-FAM |
| MyspMS10 | ACAGAACCGTAGAGGACAAAGG | | | GGGAAGAAAGAGCGGGATCA | 226-266 | 60 | | 6-FAM |
| MyspMS11 | GACTTGAGACAAGCACCCCA | ACATATAGACAAAGGGCGCCA | | | 228-261 | 60 | | 6-FAM |
| MyspMS12 | GGCAAACATGGACGGCATC | TGTGGGCGTCATGTGATGAT | | | 229-238 | 60 | | 6-FAM |
| MyspMS17 | ATACCAATTCAGCCCCCACC | AACAACGAACCCCATGTCGA | | | 223-243 | 60 | | NED |
| MyspMS18 | TAGGTGTCAGTTGAGGCGAC | GCCGAAAATTTTAGCAACATCCA | | | 232-234 | 60 | | NED |
| MyspMS19 | ATTCTGGTGCGGTGTGTCTT | GTTTCGGTCTACAACGGGGA | | | 243-245 | 60 | | NED |
| MyspMS22 | CCGAACCGACTCTCGAATGA | AGTTGAGAGGGAAGTGAGGA | | | 244-250 | 60 | | NED |
| MyspMS25 | TGGGATGCGTGAGAAAAAGT | GGAAGGATGGGAAGTGGCTT | | | 249-258 | 60 | | NED |
| MyspMS26 | ACAGGGTGTTCGTCCATGTC | AAAGGGGTAGCCAGAGTTGC | | | 257-299 | 60 | | NED |
| MyspMS27 | TCTGTGCAATGTCAGACGTT | AGAGGCCGTTTGTCTCTCAA | | | 250-262 | 60 | | NED |
| MyspMS28 | GCGCTGTTCTCTGCTAATGC | TTGCAGGTTCAGAGCGTCTT | | | 260-264 | 60 | | NED |
| MyspMS30 | CGCTCCGGCTCACATATCTT | GGGTAGCACCGGAGTTGTAA | | | 262-280 | 60 | | NED |
| MyspMS31 | GCCAATGCCAATGAACCTCC | TTGGATCAGCCGAACGAAGT | | | 224-269 | 60 | | NED |
| MyspMS34 | GCCTGTCGAGCTAACCTCTC | CAATTTCAATCCCGGTCCGC | | | 258-270 | 60 | | NED |
| MyspMS35 | CCCCCAATTTTCACGCTTCC | AATTCGCATTGTCATCCGCC | | | 256-274 | 60 | | NED |
| MyspMS41 | ATTAGGGTTGGGCCAGACAC | TCAGGTTGCACGGTGAGAAA | | | 274-282 | 60 | | NED |
| MyspMS42 | AGAGAGAGCAAGGTGAGGGT | AGGCAGCAGCAGTACTTGTT | | | 244-286 | 60 | | NED |
| MyspMS51 | GCAATGTTAGCTCAGCACGG | ATTCCCAAGGCAGATGCAGT | | | 258-294 | 60 | | VIC |
| MyspMS53 | GGATGGTGCAGGGAAGACAA | TGTTGAGTTTGGAGCGTGGA | | | 279-315 | 60 | | VIC |
| MyspMS56 | GCTAGGACTCAGGTTGGTCG | CACAATCGAACGAATGCCCC | | | 303-305 | 60 | | VIC |
| MyspMS72 | TCCTTCTGATGGCAGCTAGC | ACTCACGACTTGGCAAGGAA | | | 336-348 | 60 | | PET |
| MyspMS73 | TCCTTTTTGCTTAGGCCCGA | AAACAGAAGAGGAGCGCGAT | | | 338-354 | 60 | | PET |
| MyspMS81 | GAACCGCGACGAACTAGACT | GGATAGGCAGCTCCCTTGTC | | | 342-375 | 60 | | PET |
| MyspMS87 | TCAAACCCGTACGTCTGATAC | GCAACTCGGAAAAGAACCCG | | | 349-400 | 60 | | VIC |
| MyspMS98 | CTTGATCGCGGACGTTGAAT | GTTTACGGCTCGACCATTGC | | | 445-493 | 60 | | VIC |
| MyspMS99 | CATTGCGACTTGTCAGCAGG | AGGCAAACCCCTCAGTCAAG | | | 237-247 | 60 | | VIC |

**Table S1.** Amplification information primer sequences and characteristics of 30 microsatellite loci developed from *Myriophyllum spicatum.*

**Table S2.**  Summary of population information for six M. spicatum sampling sites in South Korea. asterisks (★) were included in spatial genetic structure analyses

| Population abbreviation | | GPS | Study site  (m²) | | Sampling area (m) | Elevation (m) | Sample  Size | Disturbance types |
| --- | --- | --- | --- | --- | --- | --- | --- | --- |
| *GS | N 36°53’14.1” E 127°56’6.4” | | | - | 150 x 30 | 110 | 20 | Flood,  the use of agricultural water, etc |
| US | N 36°18’44.1” E 128°33’41.1” | | | - | 290 x 70 | 48 | 20 |  |
| NJ | N 35°03’54.2” E 126°42’1.2” | | | 934 | 48 x 18 | 75 | 20 | the use of agricultural water, etc |
| DG | N 35°51′6.0″ E 128°39′3.8″ | | | 7,310 | 110 x 40 | 68 | 20 |  |
| *KS | N 35°49’44.7” E 128°42’53.8” | | | 39,200 | 110 x 100 | 62 | 20 | Reservoir management, etc  (e.g., vegetation removal, control structure) |
| BS | N 35°09’46.3” E 128°58’18.0” | | | 9,600 | 110 x 20 | 0.1 | 20 |  |

**Table S3.** Annual water-quality metrics (March–September means, 2016–2023) for the GS and US sites where M. spicatum was sampled. pH: Potential of Hydrogen, water temperature; BOD: biochemical oxygen demand; T-N: total nitrogen; T-P: total phosphorus

| Population | Year | pH | Water temperature (°C) | BOD  (mg O₂ L⁻¹ ) | T-N  (mg N L⁻¹ ) | T-P  (mg P L⁻¹ ) |
| --- | --- | --- | --- | --- | --- | --- |
| GS | 2016 | 8.3 | 23.2 | 1.03 | 3.09 | 0.03 |
|  | 2017 | 8.5 | 21.4 | 1.27 | 3.32 | 0.04 |
|  | 2018 | 8.2 | 18.6 | 1.06 | 3.66 | 0.03 |
|  | 2019 | 8.1 | 20.4 | 1.21 | 2.61 | 0.04 |
|  | 2020 | 8.6 | 17.8 | 0.96 | 2.88 | 0.03 |
|  | 2021 | 8.5 | 17.3 | 0.97 | 3.25 | 0.04 |
|  | 2022 | 8.2 | 16.9 | 1.39 | 3.46 | 0.03 |
|  | 2023 | 8.3 | 20.6 | 1.11 | 2.45 | 0.04 |
| US | 2016 | 7.8 | 21.3 | 1.23 | 2.24 | 0.03 |
|  | 2017 | 7.9 | 20.9 | 2.43 | 2.09 | 0.03 |
|  | 2018 | 7.6 | 21.0 | 1.79 | 3.60 | 0.06 |
|  | 2019 | 7.8 | 21.3 | 2.04 | 1.57 | 0.02 |
|  | 2020 | 7.8 | 21.0 | 2.81 | 2.30 | 0.04 |
|  | 2021 | 7.7 | 21.9 | 2.29 | 3.27 | 0.07 |
|  | 2022 | 8.3 | 23.4 | 3.25 | 1.39 | 0.11 |
|  | 2023 | 8.2 | 21.8 | 2.27 | 2.60 | 0.05 |


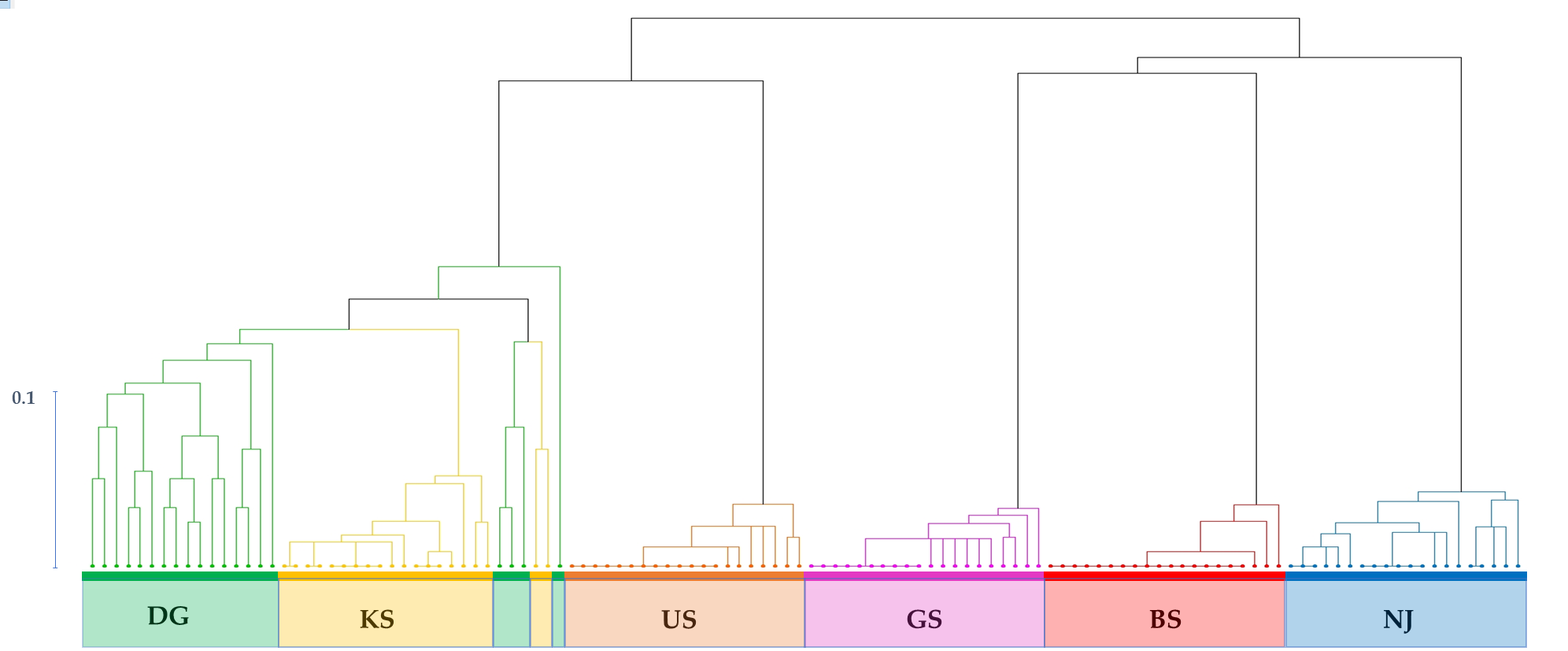


**Figure S1**. UPGMA tree based on Nei’s genetic distance, calculated for clonal and genetic relationships among six M. spicatum populations.


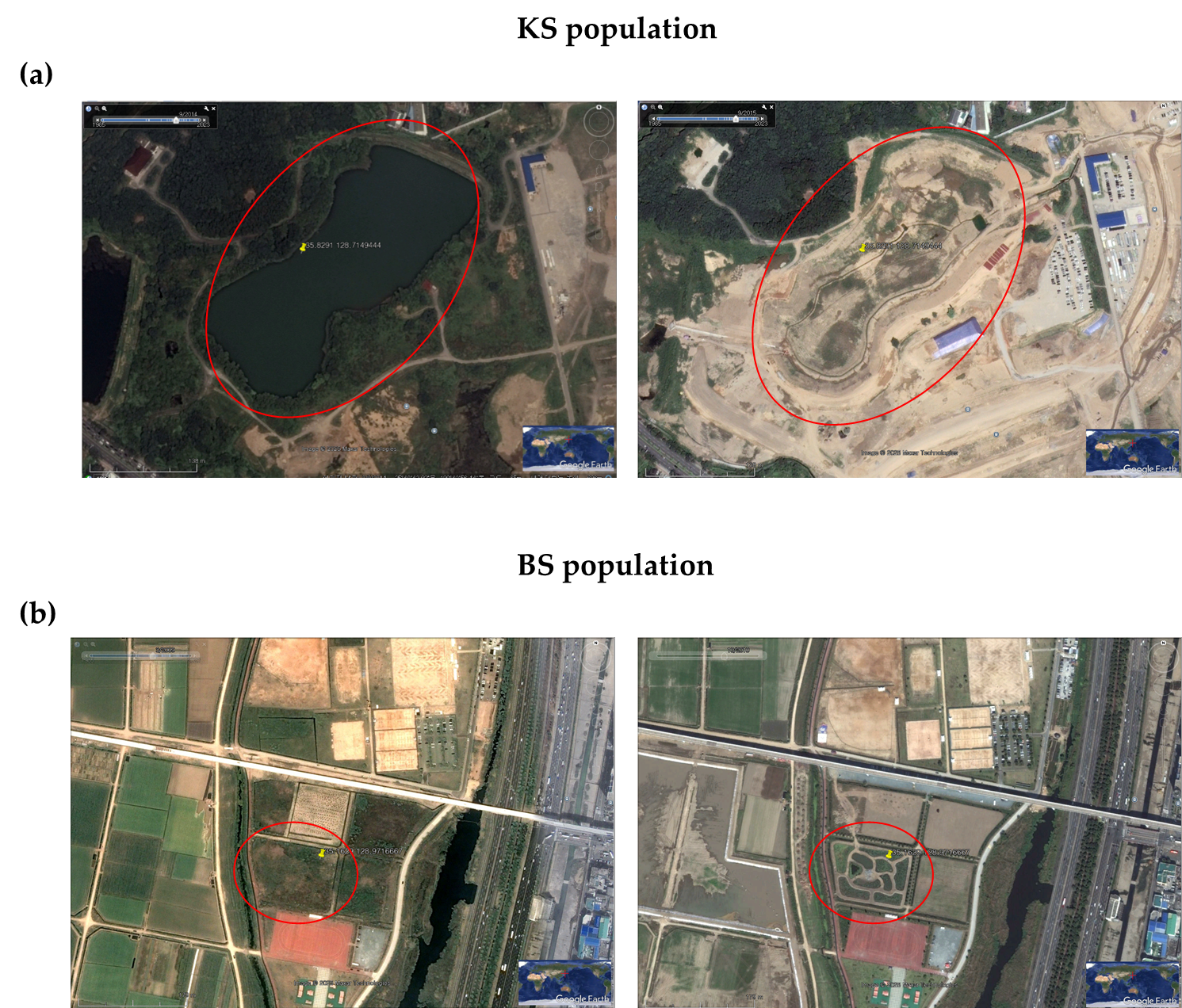
**Figure S2.** Satellite imagery showing land-use changes at two *M. spicatum* sampling sites in South Korea. (a) KS population before development (September 2014) and during lake park construction (September 2015), (b) BS population development (March 2009) and after pond establishment (October 2010).
